# Microbial modulation prevents the effects of pervasive environmental stressors on microglia and social behavior, but not the dopamine system

**DOI:** 10.21203/rs.3.rs-2548369/v1

**Published:** 2023-02-09

**Authors:** Staci Bilbo, Caroline Smith, Danielle Rendina, Marcy Kingsbury, Karen Malacon, Dang Nguyen, Jessica Tran, Benjamin Devlin, Ravikiran Raju, Madeline Clark, Lauren Burgett, Jason Zhang, Murat Cetinbas, Ruslan Sadreyev, Kevin Chen, Malvika Iyer

**Affiliations:** Duke University; Duke University

## Abstract

Environmental toxicant exposure, including air pollution, is increasing worldwide. However, toxicant exposures are not equitably distributed. Rather, low-income and minority communities bear the greatest burden, along with higher levels of psychosocial stress. Both air pollution and maternal stress during pregnancy have been linked to neurodevelopmental disorders such as autism, but biological mechanisms and targets for therapeutic intervention remain poorly understood. We demonstrate that combined prenatal exposure to air pollution (diesel exhaust particles, DEP) and maternal stress (MS) in mice induces social behavior deficits only in male offspring, in line with the male bias in autism. These behavioral deficits are accompanied by changes in microglial morphology and gene expression as well as decreased dopamine receptor expression and dopaminergic fiber input in the nucleus accumbens (NAc). Importantly, the gut-brain axis has been implicated in ASD, and both microglia and the dopamine system are sensitive to the composition of the gut microbiome. In line with this, we find that the composition of the gut microbiome and the structure of the intestinal epithelium are significantly shifted in DEP/MS-exposed males. Excitingly, both the DEP/MS-induced social deficits and microglial alterations in males are prevented by shifting the gut microbiome at birth via a cross-fostering procedure. However, while social deficits in DEP/MS males can be reversed by chemogenetic activation of dopamine neurons in the ventral tegmental area, modulation of the gut microbiome does not impact dopamine endpoints. These findings demonstrate male-specific changes in the gut-brain axis following DEP/MS and suggest that the gut microbiome is an important modulator of both social behavior and microglia.

## Introduction

Air pollution represents a global and ever-increasing burden to human health^1,2^. Importantly, there are pervasive socioeconomic disparities in air pollution exposure. Low-income and racial minority communities are exposed to higher levels of air pollution than high socioeconomic status, primarily white, communities^3^. These communities are also subjected to fewer resources and higher levels of psychosocial stress^4^. This convergence of toxicant and psychosocial burdens makes these populations more susceptible to the adverse health consequences of air pollution exposure^5^.

High levels of air pollution, particularly during development, have been linked to several diseases, including autism spectrum disorder (Odds ratios: 1.98–3.48, Hazard ratios: ~1.14)^6,7,8^. Like air pollution, gestational maternal stress is associated with an increased risk of having a child with ASD^9,10^. ASD is a neurodevelopmental disorder primarily characterized by deficits in social behavior/communication, as well as repetitive behaviors. ASD is also characterized by a strong male bias in prevalence (4:1) and males tend to be more sensitive than females to early life stressors^11,12^. Together, these findings suggest that air pollution and maternal stress during pregnancy may synergize to increase ASD risk, particularly in male offspring.

Many brain regions have been implicated as disrupted in function and/or connectivity in ASD, including the mesolimbic reward circuit^13,14,15^. Activity in this circuit is critical to reward/motivation and social interactions. Optogenetic stimulation of nucleus accumbens (NAc)-projecting dopamine neurons in the ventral tegmental area (VTA) increases social behavior in mice^16^ and dopaminergic signaling in the NAc mediates social play behavior in rodents^17,18^. Microglia, the resident immune cells of the brain, respond potently to air pollution^19^ and play a critical role in the developmental organization of the dopamine system^17,20^. However, little is known regarding the impact of prenatal environmental exposures on microglia or dopaminergic function within this pathway.

The gut-brain axis has emerged as key to the pathophysiology and potential treatment of ASD^21^. Changes in the composition of the gut microbiome and gastrointestinal symptoms have been documented in individuals with ASD and long-lasting improvements in both gastrointestinal and behavioral symptoms are found following microbiota transfer therapy^22,23^. Changes in gut microbiota in ASD may be driven by the highly restrictive diets of individuals with ASD^24^, but regardless of the source of these changes, the gut microbiome represents an important site for therapeutic intervention in the amelioration of behavior impairments in this disorder.

Importantly, several studies suggest that microglial function and activity of the dopamine system are impacted by the composition of the gut microbiome. For instance, germ-free mice (lacking microbiota) have immature, hyper-ramified microglia^25,26^. In mouse models of ASD, supplementation with the bacterial species *Lactobacillus reuteri* induced neuroplasticity in dopaminergic neurons and restored sociability^27,28^. While studies have shown that air pollution shifts the composition of the gut microbiome in adulthood^29,30^, nothing is known about how maternal exposure to air pollution during gestation might impact the gut microbiome of offspring, and how these shifts might impact the development of circuits underlying social behavior.

To examine these questions, our lab established a paradigm in which mouse dams are exposed to combined diesel exhaust particles (DEP) and maternal stress (MS) during pregnancy^31,32^. We report that maternal DEP/MS alters microglial morphology and gene expression and decreases dopaminergic tone in the NAc only in male offspring. We show for the first time that prenatal DEP/MS alters the composition of the gut microbiome in male offspring only, and that shifting the colonization of the gut microbiome at birth via a cross-fostering procedure prevents both sociability deficits and changes in microglial morphology in DEP/MS-exposed males. Interestingly, while chemogenetic activation of the dopamine system is sufficient to rescue social behavior deficits following DEP/MS in males, microbial modulation at birth does not appear to impact the dopamine system, suggesting that prevention of microglial phenotypes is more closely associated with the prevention of social behavior deficits following DEP/MS.

## Methods

### Animals

Wild-type (WT) *C57Bl/6J* mice were purchased from Jackson Laboratories (Stock # 000664). DAT-IRES-Cre mice (Jackson Laboratories, Stock # 006660) were obtained from Dr. Henry Yin at Duke University. Animals were group-housed under standard laboratory conditions (12-hour light/dark cycle [6am-6pm], 23°C, 60% humidity). To account for litter effects, offspring were obtained from multiple litters in all treatment groups/experiments (Supplementary Table 1 for complete litters and animals). Experiments were conducted in accordance with the NIH *Guide to the Care and Use of Laboratory Animals* and approved by the Massachusetts General Hospital Institutional Animal Care and Use Committee (IACUC), and subsequently, the Duke University IACUC.

### DEP/MS exposures

#### DEP instillations

Diesel exhaust particles (DEP) were obtained from Dr. Ian Gilmour at the Environmental Protection Agency and exposures were conducted in accordance with Bolton et al., 2013^31^. Multiparous C57Bl/6J females were time mated. A vaginal plug was taken as an indication of pregnancy and set as embryonic day (E)0. Females were then pair-housed based on embryonic day until E13. Instillations occurred every 3 days throughout pregnancy, beginning on E2, totaling 6 instillations. Briefly, females were weighed, anesthetized with 2% isoflurane, and administered either 50 μg/50 μL of DEP in vehicle (0.05% Tween20 in PBS; dose previously validated/published^19,31,32^), or vehicle (CON) via oropharyngeal instillation during which females are suspended by their incisors from a plastic wire and the instillation is administered over the course of 30 sec. Females were monitored until they were awake before returning to their home cages.

#### Maternal Stress (MS)

To induce prenatal maternal stress, we adapted a nest restriction paradigm^19,33^. On E13, females were singly housed and placed into a cage containing a thin layer of AlphaDri bedding (AlphaDri; Shepherd Specialty Papers) covered by an elevated aluminum mesh platform (0.4 cm × 0.9 cm mesh; McNichols Co., Tampa, FL) and given 2/3 (~ 1.9 grams) of a cotton nestlet (MS condition) or placed into a cage with AlphaDri bedding and an entire nestlet (CON condition). On E18.5 (evening before delivery), dams were transferred to a clean cage of AlphaDri bedding with a full nestlet. Offspring were weaned into cages with same sex littermates at postnatal day (P)24, as recommended by our IACUC policy.

#### Behavioral Testing

Behavioral testing took place between postnatal days P27-P40, during the second half of the light phase (afternoon). For all assays, males and females were tested using separate apparatuses on different days. Animals were handled and habituated to the testing room and testing apparatus prior to testing.

#### Sociability and Social Novelty Preference

To assess sociability (preference to investigate a social vs a non-social stimulus) and social novelty preference (novel social stimulus vs. cage mate) a 3-chambered social preference test was used^34^. This test consists of a 3-chambered arena with openings allowing passage between the chambers. Stimuli were confined within smaller containers (Plexiglass rod sides) in each of the side chambers. Subject animals were placed into the middle chamber and freely allowed to investigate each stimulus over the course of 5 min (sociability) or 10 min (social novelty preference; see Supplementary Methods for full details).

#### Marble Burying

To assess repetitive behavior, mice were placed into clean mouse cages with 5cms of wood shavings and 20 blue and black marbles arranged in a 4×5 grid. Each cage was photographed prior to introducing the subject mouse. 20 min. later, each mouse was removed, and a second picture was taken, from which the number of buried marbles was counted by a blind observer. Marbles were considered “buried” if more than 2/3 of their surface was no longer visible.

#### Open Field

To assess ‘anxiety-like’ behavior, mice were placed into an open arena (45cm × 45cm). A central square within the arena was outlined on the bottom of the test (15cm × 15cm). Ethovision software (Noldus) was used to quantify total distance traveled, velocity, and time in the central square for 10 min.

#### Microglial RNA sequencing

To isolate microglia for RNA sequencing, bilateral NAc was micro-dissected from saline perfused brains at P60 and microglial isolations were conducted using a CD11b antibody-based procedure, according to published methods^35,36^. RNA was extracted from both CD11b+ (microglia) and CD11b-cell populations. Samples were transferred to the MGH Next Generation Sequencing Core for sequencing on an Illumina HiSeq 2500 instrument and the resulting data was analyzed in house using DESeq2^39^, WebGestaltR (version 0.4.4), RRHO2 (version 1.0), and custom scripts (see Supplementary Methods^37–40^).

#### Immunohistochemistry (IHC)

For all IHC, animals were euthanized via CO_2_ inhalation during adolescence (P30–45) and brains were perfused with ice-cold saline followed by 4% paraformaldehyde. Brains were then removed and post-fixed for 48 hrs in 4% paraformaldehyde, followed by 48 hrs in 30% sucrose with 0.1% sodium azide. Next, brains were flash frozen in 2-methylbutane and stored at −20°C until sectioning at 40μm on a cryostat (Leica Biosystems). All IHC methods were conducted according to our previous work^32^, and detailed methods, antibody concentrations, and imaging procedures can be found in Supplementary methods.

### Tissue Punches for Gene Expression

#### Tissue Collection and Punches

At P45, animals were euthanized and perfused with ice cold saline. Brains were removed and frozen in 2-methylbutane. Punches were collected using a 1mm diameter core sampling tool (Electron Microscopy Sciences). Brains were mounted in a sterilized cryostat and punches were collected by inserting the sterilized core sampling tool to a depth of 1mm. Punches were immediately placed into Trizol and frozen at −80°C until RNA extraction. RNA was extracted, cDNA synthesized, and qPCR run on a Mastercycler ep realplex (Eppendorf). qPCR primers were designed in house and purchased from Integrated DNA technologies. Relative gene expression was calculated using the 2-ΔΔCT method, relative to the house-keeping gene (*18S*) and the lowest sample on the plate (see Supplementary Methods).

#### Chemogenetic manipulations

DAT-Ires-Cre homozygous males were bred with WT *C57Bl/6* females which were then exposed to DEP/MS or CON during pregnancy. Resulting offspring were heterozygous (Cre+) and underwent stereotaxic surgery on P23–24 to virally inject either pAAV-hSyn-DIO-hM3D(Gq)-mCherry (Excitatory DREADD; Addgene #44361, titers: 7×10^12^ vg/ml) or pAAV-hSyn-DIO-mCherry (Control mCherry reporter; Addgene #50459, titers: 7×10^12^ vg/ml) into the VTA. Coordinates were, AP: − 2.6, ML +/−1.2, DV – 4.8, 10°angle (Adapted from Franklin & Paxinos Mouse Brain Atlas^41^ for use in juveniles). See Supplementary methods for complete procedural details. On P33–35, subjects were injected with either Vehicle (1% DMSO in sterile saline) or CNO (1mg/kg in 1% DMSO in sterile saline) 30 min. prior to sociability testing. After testing, all animals were euthanized, and brains were collected to verify transfection of the VTA.

#### 16S Microbiome sequencing

Bacterial taxa were identified using 16S rRNA amplicon sequencing of microbiome samples. Library preparation and sequencing were conducted in accordance with standard protocols (earthmicrobiome.org) on an Illumina MiSeq Instrument. Qiime2–2020.2 analysis platform, PAST (PAleonto- logical Statistics^45^), and the R environment (version 3.4.2) were used to analyze 16S data (see Supplementary Methods^46,47,48,49,50^). Microbiome sequencing was conducted on cecal microbiome samples collected from both dams and offspring, as well as on maternal vaginal lavages and milk samples (see Supplementary Methods).

#### Gut tissue processing

Following euthanasia via CO_2_ inhalation and perfusion with ice-cold saline, intestines were removed and placed into a petri-dish of sterile 1X PBS. ~1.5cm segments from the duodenum (proximal to stomach), and ileum and colon (proximal to cecum) were collected. Segments were gently compressed to clear contents, and a) flash frozen in liquid nitrogen for mRNA, b) postfixed for 48 hr in 4% paraformaldehyde followed by 30% sucrose for IHC, or c) postfixed for 5 days in 4% paraformaldehyde followed by 70% Ethanol for H&E staining (Supplementary Methods).

#### Cross-fostering experiments

For cross-fostering experiments, WT C57Bl/6 dams were mated and exposed to CON or DEP/MS as described. On P0, pups were cross-fostered to dams of the same treatment (CON-CON [C→ fC] and DEP/MS-DEP/MS [D→ fD]), or to dams of the opposite treatment (DEP/MS-CON [D→ fC] and CONDEP/MS [C→ fD]). Entire litters were swapped by placing them in a small cup filled with bedding from the recipient mother. The entire litter was then placed into the recipient cage at once. Maternal care was assessed 3x daily for the first 3 days of life (Supplementary Methods). Offspring were weaned at P24 into cages with same sex littermates. Offspring behavioral and neural endpoints were assessed according to the methods described above and in Supplementary Methods.

#### Statistics

Statistical analyses were conducted using GraphPad Prism 9 software, the Qiime2–2020.2 analysis platform, PAST (PAleonto-logical STatistics), the R environment, and Python. Detailed descriptions of all statistical methods are available in the figure legends and in Supplementary Table 2. Data are expressed as mean +/− SEM and statistical significance was set at p < 0.05.

## Results

### Prenatal DEP/MS exposure induces social deficits in male offspring only

We characterized social behavior in offspring following prenatal exposure to either combined DEP/MS (diesel exhaust particles and maternal stress) or control (CON) conditions. No group differences were observed in maternal weight gain during pregnancy, litter size, sex ratio, or offspring body weight (Fig. S1). For complete litters, animal numbers, and statistics throughout the manuscript, see figure legends and Supplementary Tables 1 & 2, respectively).

**Figure 1:**
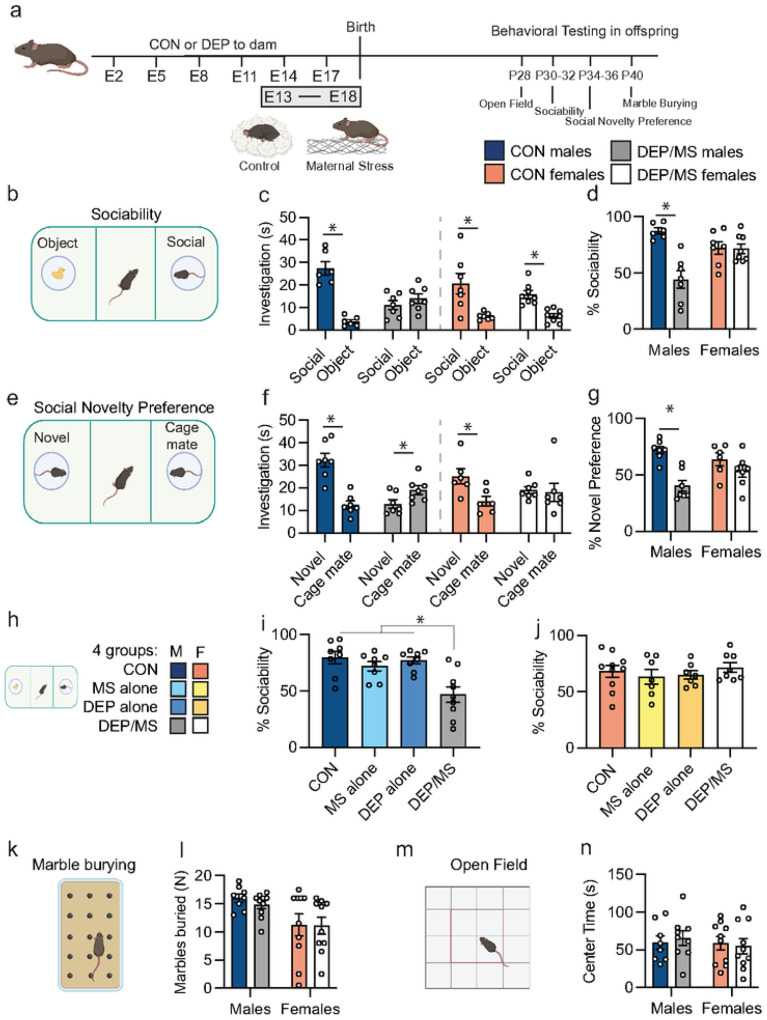
DEP/MS impairs social behavior in male but not female offspring. **a**, Schematic of DEP/MS or CON procedure. **b**, Sociability assay. **c**, Social investigation time is significantly higher than object investigation time in all groups except DEP/MS males (N=6–8/group, un-paired t-tests [social vs. object]: CON M: p<0.001, DEP/MS M: p=0.32, CON F: p<0.01, DEP/MS F: p<0.001). **d**, Social preference is significantly lower in DEP/MS males as compared to CON males. However, DEP/MS does not alter social preference in females (N=6–8/group; 2-way ANOVA [treatment × sex], treatment: p<0.001, sex: p=0.28, interaction: p<0.001, Bonferonni posthoc: CON male vs. DEP/MS male: p<0.001). **e**, Social Novelty Preference Test. **f**, CON males and females significantly prefer a novel social stimulus over a cage mate. DEP/MS males significantly prefer a familiar cage mate, while DEP/MS females show no preference (N=6–8/group, un-paired t-tests [novel vs. cage mate]: CON M: p<0.001, DEP/MS M: p=0.04, CON F: p=0.02, DEP/MS F: p=0.84). **g**, Social novelty preference is significantly lower in DEP/MS males as compared to CON males. However, DEP/MS does not alter social novelty preference in females (N=6–7/group; 2-way ANOVA [treatment × sex], treatment: p<0.001, sex: p=0.23, interaction: p=0.04, Bonferonni posthoc: CON male vs. DEP/MS male: p<0.01). **h**, Schematic of all four groups design **i**, In male offspring, neither DEP or MS alone impairs sociability (N=8–10/group; one-way ANOVA [treatment], treatment p<0.001) **j**, Neither DEP alone, MS alone, nor DEP/MS alters sociability in females (N=7–10/group; one-way ANOVA [treatment], treatment p=0.71). **k**, Marble-burying. **l**, DEP/MS had no effect on marble burying behavior (N=8–10/group; 2-way ANOVA [treatment × sex], treatment: p=0.63, sex: p=0.004, interaction: p=0.71). **m**, Open Field. n, DEP/MS has no effect on center time in the open field (N=8–10/group; 2-way ANOVA [treatment × sex], treatment: p=0.90, sex: p=0.55, interaction: p=0.60). Data represent mean + SEM, *p<0.05. CON: vehicle/control, DEP/MS: diesel exhaust particles/maternal stress. M: males, F: females, E: Embryonic, P: postnatal.

The adolescent period – ~ postnatal day (P) 25–45 in mice – is one during which social interactions with peers are of heightened importance. Thus, we conducted behavioral testing during this period (Fig. 1a). In the sociability assay, we found that CON males showed a strong preference for a novel sex-, age-, and treatment- matched social stimulus as compared to an object, whereas DEP/MS males did not (Fig. 1b–d). This effect was male-specific as no such difference was observed in females. In the social novelty preference test, CON males showed a strong preference for a novel social stimulus over a cage mate, while DEP/MS males did not (Fig. 1e–g). To ascertain whether social deficits were driven primarily by one prenatal exposure or the other, we tested sociability in offspring following DEP or MS alone. Neither treatment on its own induced social deficits, indicating that synergism between the two is required (Fig. 1h–j). Finally, we observed no treatment effects on marble-burying (Fig. 1k&l) or anxiety-like behavior (Fig. 1m&n), suggesting that the effects of DEP/MS are specific to the social domain, at least during adolescence.

### DEP/MS induces a hyper-ramified phenotype in male but not female microglia

**Figure 2:**
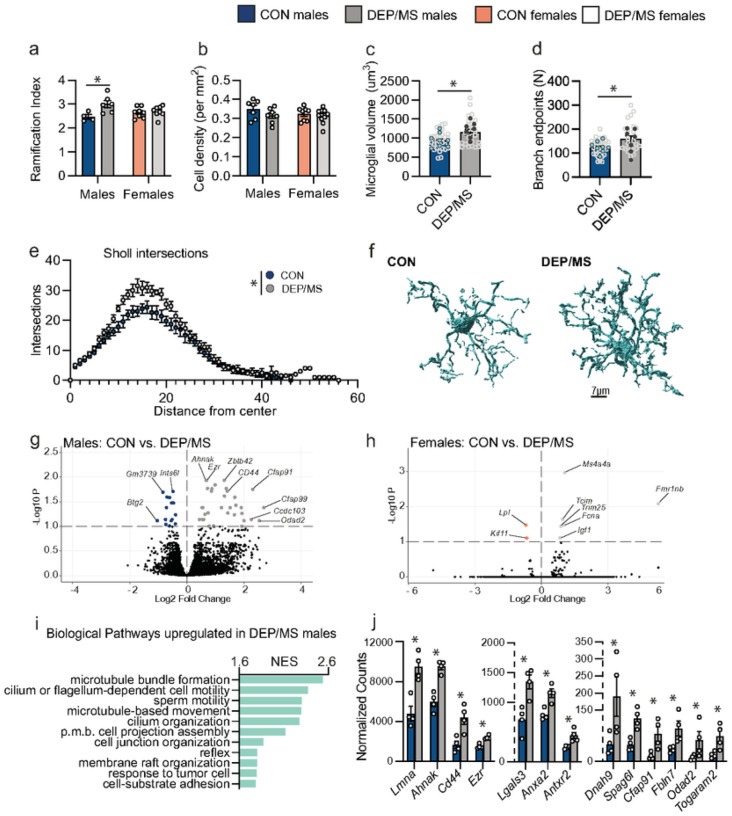
DEP/MS induces a hyper-ramified phenotype in male but not female microglia. **a**, Male, but not female, microglia are hyper-ramified following DEP/MS, as assessed using 3DMorph (N=4–8 animals/group, avg. 11 microglia analyzed/mouse, 2-way ANOVA [treatment × sex], treatment: p=0.012, sex: p=0.55, interaction: p=0.03, Bonferonni posthoc: CON vs DEP/MS males: p=0.02). **b**, No differences were observed between CON and DEP/MS in cell density (N=7–9 animals/group, 2-way ANOVA [treatment × sex], treatment: p=0.12, sex: p=0.35, interaction: p=0.43) **c**, Imaris 3D reconstruction revealed larger microglial volume following DEP/MS in males, as well as more branchpoints (**d**) and more sholl intersections (**e**)(N=25–30 microglia from 7–8 animals, nested t-tests [CON vs DEP/MS], volume: p=0.02, branch endpoints: p=0.03, sholl intersections: p=0.02). In c and d, pale grey dots represent individual microglia while darker dots represent animal averages. **f**, Representative reconstructions of CON and DEP/MS microglia, scale=7μm. **g**, Volcano plots of microglia gene expression reveal numerous genes that are differentially expressed in males following DEP/MS at an adjusted p value cutoff of 0.1 (N=4/group, positive Log2 fold change indicates higher expression following DEP/MS. **h**, In contrast, very few genes are differentially expressed following DEP/MS in females at the same cutoff (N=4/group, positive Log2 fold change indicates higher expression following DEP/MS). **i**, GSEA analysis demonstrates up-regulation in pathways related to cell projection assembly and motility in males following DEP/MS (N=4/group, all pathways reached significance with FDR p<0.05). **j**, Genes related to cell motility and process extension/remodeling were up-regulated in males following DEP/MS. Data represent mean +/− SEM in a-e & j, *p<0.05. CON:vehicle/control, DEP/MS: diesel exhaust particles/maternal stress, N: number, NES: normalized enrichment score, p.m.b.: plasma membrane bound, GSEA: gene set enrichment analysis.

Developmental insults have been shown to have a particularly potent impact on microglia, the resident immune cells of the brain. Microglia play a critical role in the organization of neural circuits via synaptic pruning^17^, trogocytosis^19^, and other neuronal contracts^19^. Gestational DEP increases microglial-neuronal interactions in cortex, and DEP/MS impairs microglial pruning of thalamocortical synapses in male offspring^31,32^. Therefore, we tested how DEP/MS impacts microglia in the NAc. Microglial morphology is often taken as an early indicator of alterations in microglial function. Using a MATLAB-based, semi-automatic program to quantify microglial ramification (3DMorph^52^), we found that NAc-microglia are hyper-ramified following DEP/MS in males but not in females (Fig. 2a). No difference was observed in microglial density between CON and DEP/MS males or females, assessed using IHC for Iba1 (quantified by cell density; Fig. 2b). To further define changes in microglial morphology in males, we used Imaris 3D image reconstruction software which revealed increased total volume of NAc-microglia following DEP/MS (Fig. 2c). Sholl analysis revealed significantly more branch endpoints and Sholl intersections in DEP/MS male microglia as compared to CON (Fig. 2d–f). In females, no treatment effects were observed on microglial volume, branch endpoints, or Sholl intersections (Fig. S2). These findings demonstrate, using multiple approaches, that male microglia are larger and hyper-ramified in the NAc following DEP/MS.

To gain a deeper understanding of how DEP/MS affects microglial function, we performed RNA sequencing of NAc-microglia. Notably, we found more differentially expressed genes (DEGs) in male microglia following DEP/MS (49; Fig. 2g) than in female microglia (8; Fig. 2h). In males, more genes were significantly up- (34) than down- (15) regulated following DEP/MS. In keeping with the hyper-ramification observed, gene set enrichment analysis (GSEA) revealed enrichment of biological and cellular pathways involved in cell motility, extracellular matrix interactions, and cell remodeling/projection assembly in male microglia following DEP/MS (Fig. 2i, Fig. S2). For example, several of the most highly and differentially transcribed genes encode proteins critical for cell motility/migration (*Ahnak, Dnah9, Odad2, Togaram2, Cfap91, Cfap99*), extracellular matrix interactions and cell adhesion (*CD44, Antxr2, Fbln7, Lgals3*), and intracellular remodeling (*Lmna, Ezr, Syne3, Spag6l*; Fig. 2j). Interestingly, GSEA analysis revealed a distinct set of biological pathways that were enriched in female microglia following DEP/MS, and no such changes in non-microglial cells (CD11b- population; Fig. S2).

### DEP/MS alters dopamine circuitry in males but not females

**Figure 3:**
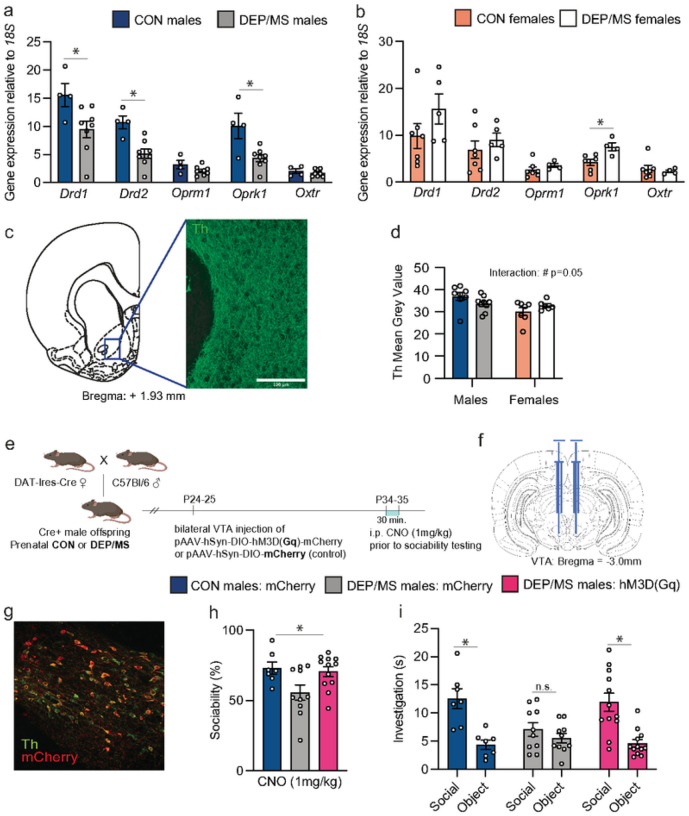
Chemogenetic activation of VTA dopamine neurons rescues sociability in DEP/MS males. **a**, Gene expression in the NAc at P45 for socially relevant receptors in males. mRNA for the dopamine D1 receptor (Drd1), D2 receptor (Drd2) and the kappa opioid receptor (Oprk1) are reduced following DEP/MS in males, while mu opioid receptor (Oprm1) and oxytocin receptor (Oxtr) mRNA are not (N=4–8/group, unpaired t-tests [CON vs DEP/MS], Drd1: p=0.036, Drd2: p=0.003, Oprm1: p=0.12, Oprk1: p=0.008, Oxtr: p=0.48). **b**, DEP/MS increases Oprk1 mRNA in females as compared to CON, but not mRNA for Drd1, Drd2, Oprm1, or Oxtr (N=4–7/group, unpaired t-tests [CON vs DEP/MS], Drd1: p=0.19, Drd2: p=0.43, Oprm1: p=0.32, Oprk1: p=0.017, Oxtr: p=0.66). **c**, Representative images of tyrosine hydroxylase (Th) fiber density in the NAc. **d**) Trend towards a significant interaction effect for Th mean grey value in the NAc with lower signal in DEP/MS males as compared to CON, but higher in females at P45 (N=4–9/group, 2-way ANOVA [treatment × sex], treatment: p=0.81, sex: p=0.016, interaction: p=0.053; CON vs. DEP/MS males: p=0.12; females: p=0.22). **e**, Experimental timeline: Following prenatal DEP/MS, Dat-Cre+ male CON and DEP/MS males underwent stereotaxic microinjection of DREADD virus (Gq [excitatory] or mCherry [control] at PND23–24. At P33–35, social behavior was tested 30 min. after i.p. CNO injection. **f**, Mouse brain atlas image of location of microinjection. **g**, Representative 20x image of dopamine bers (Th) co-labeled with mCherry in the VTA. **h**) Social preference is significantly increased in DEP/MS males following chemogenetic activation of dopaminergic cells in the VTA (N=7–12/group, one-way ANOVA [treatment], p=0.025). **i**, Chemogenetic activation of dopaminergic cells in the VTA restores social preference (N=7–12/group, unpaired t-test [social vs. object], p<0.001. Data are represented as mean + SEM, *p<0.05, #p=0.05. VTA: ventral tegmental area, NAc: nucleus accumbens, Th: tyrosine hydroxylase, P: postnatal, CON: vehicle/control, DEP/MS: diesel exhaust particles/maternal stress, i.p.: intraperitoneal, CNO: Clozapine-N-oxide, DREADD: Designer Receptors Exclusively Activated by Designer Drugs.

Based on our microglial findings, we hypothesized that NAc-microglia might be interacting more with neuronal populations in the NAc, potentially to alter social behavior. Indeed, microglia eliminate dopamine D1 receptors (D1Rs) in the NAc (with a specific peak during adolescence [at P30]), and this D1R elimination is critical to the developmental trajectory of social play^18^. Dopamine, endogenous opioids, and oxytocin all mediate social motivation by acting within the NAc^18,53,54^. Thus, we performed tissue punching and qPCR for mRNA for dopamine receptors (*Drd1* and D*rd2*), opioid receptors (*Oprk1*, *Oprm1*) and oxytocin receptor (*Oxtr*). We found that both *Drd1* and *Drd2* mRNA were decreased in the NAc of adolescent male offspring following DEP/MS exposure (Fig. 3a), but not in females (Fig. 3b). mRNA was not decreased for Oprm1 or *Oxtr* although, interestingly, Oprk1 mRNA was lower in males and higher in females (Fig. 3a&b). No changes in these receptors were observed in the amygdala (Fig. S3) – which is also a critical regulator of social behavior via connections with the NAc^55^.

We next asked whether decreases in D1R might be due to increased microglial phagocytosis of D1R at P30. However, we observed no differences in microglial engulfment of D1R using IMARIS 3D volumetric reconstructions of D1R and microglia (Iba1; Fig. S3). Alternatively, reduced dopaminergic input from the VTA might account for the lower expression of NAc-D1/D2Rs following DEP/MS in males. Therefore, we used immunohistochemistry to quantify tyrosine hydroxylase (Th) fiber density within the NAc as a measure of dopaminergic input. We observed a significant interaction effect whereby Th mean grey value (a measure of density) was decreased in males but increased in females following DEP/MS (Fig. 3c&d). These data show that decreased dopamine receptor expression in DEP/MS males coincides with a decrease, albeit small, in dopamine input from the VTA into the NAc.

### Chemogenetic activation of the dopamine system rescues male social deficits following DEP/MS

Given the reduced Th-fiber density in the NAc in male offspring following DEP/MS, we tested whether chemogenetic activation of the dopamine system would rescue social deficits in male offspring following DEP/MS. A Dat-Ires-Cre mouse line was used to generate offspring expressing Cre under the control of the dopamine transporter 1 (DAT) promotor (Fig. 3e). Cre + offspring were exposed to either CON or DEP/MS prenatally, and subsequently underwent stereotaxic viral transfection (P24–25) and social behavior testing (during adolescence; Fig. 3f–g). We found that CON males transfected with the control virus and treated with CNO showed a significant preference for the social stimulus, as we have previously observed (Fig. 3h). As predicted, DEP/MS males transduced with the control virus and treated with CNO displayed no social preference (Fig. 3h). In contrast, DEP/MS males transduced with the excitatory DREADD receptor showed a reinstatement of their preference for a social stimulus following CNO administration (Fig. 3h). Both CON males transduced with a control virus and DEP/MS males transduced with the excitatory DREADD virus spent significantly more time investigating the social stimulus as compared to an object, while DEP/MS males transduced with the control virus spent equal amounts of time investigating the animal and the object (Fig. 3i). Together, these findings suggest that activating VTA-dopamine neurons is sufficient to restore sociability following prenatal DEP/MS in males.

### DEP/MS shifts the composition of the gut microbiome and epithelium

Our results so far demonstrate male-specific changes in social behavior, microglial hyper-ramification, and decreased dopaminergic tone within the NAc following DEP/MS. Importantly, changes in all these endpoints are observed following gut microbiome manipulation. In multiple ASD mouse models, supplementation with *L. reuteri* rescues social behavior deficits by modulating activity of the dopamine system^27,28^. Microglia are also exquisitely sensitive to gut microbiota^25,26^. Microglial hyper-ramification, very similar to that observed in DEP/MS males, is reported in germ-free mice^25^.

**Figure 4:**
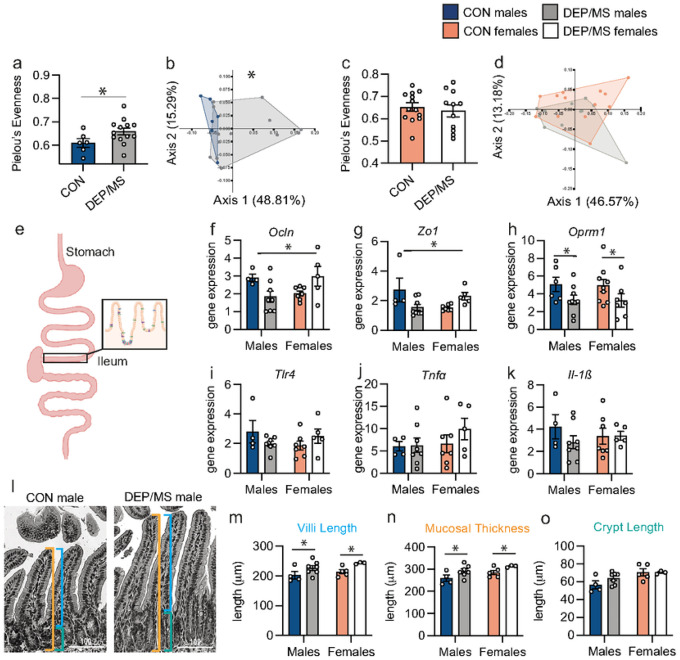
DEP/MS shifts the composition of the gut microbiome and epithelial gene expression and anatomy. **a**, Pielou’s evenness was significantly higher in DEP/MS males as compared to CON (N=6–13/group, p=0.02). **b**, CON and DEP/MS males clustered distinctly in PCoA analyses (N=6–13/group, weighted UniFrac dissimilarity, p=0.03). **c-d**, No changes in alpha or beta diversity were observed in females following DEP/MS exposure (N=11–13/group, Pielou’s evenness: p=0.66, weighted UniFrac dissimilarity: p=0.29). **e**, Representative image of section of the ileum assessed. **f-g**, DEP/MS exposure decreases tight-junction mRNA (Ocln, **f**; Zo1, **g**) in males, but increases expression in females (N=4–8/group, 2-way ANOVA [treatment × sex], Ocln: interaction: p<0.01, Zo1: interaction: p<0.01). h, DEP/MS decreases Oprm1expression in both males and females as compared to CON (N=6–9/group, 2-way ANOVA [treatment × sex], treatment: p=0.02). **i-k**, DEP/MS has no effect on proinflammatory gene expression in the ileum (Tlr4, **i**; TNFα, **j**; Il-1β, N=4–8/group, 2-way ANOVA [treatment × sex], Tlr4: treatment: p=0.82, TNFα: treatment: p=0.38, Il-1β: treatment: p=0.36). **l**, Representative H&E staining of epithelium within the ileum. lines: orange=mucosal thickness, blue= villus length, grey=crypt length. Scale=100μm. **m**, DEP/MS increases villi length and mucosal thickness (**n**) in both males and females as compared to CON but has no effect on crypt length (**o**; N=4–7 animals/group, 2-way ANOVA [treatment × sex], villi length: treatment: p=0.01, mucosal thickness: treatment: p=0.01, crypt length: treatment: p=0.24). Data represent mean + SEM, *p<0.05. CON: control, DEP/MS: diesel exhaust particles/maternal stress.

Based on these findings, we asked whether DEP/MS impacts the gut microbiome in offspring using bacterial 16S sequencing of cecal contents in offspring during early adulthood (P45). Measures of alpha diversity quantify community richness (how many bacterial taxa are present) or evenness (how evenly abundant the taxa are that form the community) within the gut microbiome of an individual animal. Community evenness was significantly increased in DEP/MS males as compared to CON (Pielou’s evenness; Fig. 4a). Principal Coordinate Analysis (PCoA) of quantitative beta diversity indices revealed distinct clustering of microbiome profiles in CON and DEP/MS males (Fig. 4b). Significant differences were also observed in the phylogenetic relatedness and abundance of microbial communities between CON and DEP/MS males (Permutational multivariate analysis of variance [PERMANOVA]). Differences were not evident at the level of individual taxa. Notably, no changes in either alpha or beta diversity were found in female offspring (Fig. 4c&d).

Microbes within the gut interface directly with the intestinal epithelium and are important determinants of epithelial structure and immunity. The tight-junction proteins Occludin (*Ocln*) and Zonula occludins-1 (*Zo1*) stabilize the gap junctions between epithelial cells. Gastrointestinal dysfunction and evidence for disruption of the gut epithelial barrier – including changes in *Ocln* and *Zo1* expression – are reported in patients with ASD^56,57^. We observed a sex-specific effect of DEP/MS (decreased in males but increased in females) on *Ocln* and *Zo1* mRNA in the ileum (Fig. 4e–g) and duodenum (Fig. S4), but not the colon (Fig. S4). Constipation and diarrhea are predominant components of GI dysfunction in ASD^56^. *Oprm1* mRNA – a critical regulator of gut motility - was reduced following DEP/MS (Fig. 4h). Interestingly, we observed no changes in the proinflammatory genes *Tlr4*, *Tnfα*, or *Il-1β* in either the ileum (Fig. 4i–k) or colon (Fig. S4), suggesting that these effects are not due to current in ammation, per se. The structure of the intestinal epithelium itself is also sensitive to microbial composition^58,59,60^. Villi length (Fig. 4l–m) and mucosal thickness (Fig. 4n), but not crypt length (Fig. 4o), were increased following DEP/MS exposure. Together, these findings demonstrate pervasive, male-biased changes in the microbiome and intestinal epithelium following DEP/MS – suggestive of decreased gut barrier function specifically in male offspring.

### DEP/MS shifts microglial gene expression towards a germ-free phenotype

**Figure 5:**
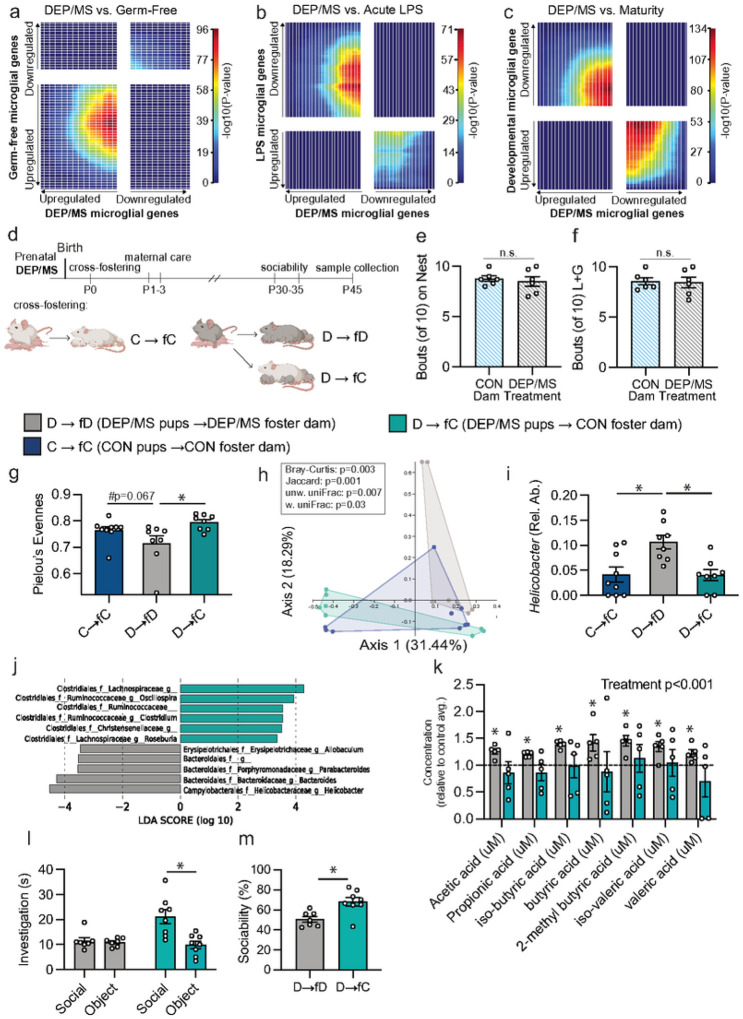
Cross-fostering at birth prevents DEP/MS-induced social deficits in male offspring. **a-c**, Strati ed Rank-Rank Hypergeometric Overlap (RRHO) analysis on RNA sequencing of isolated microglia from the NAc of male offspring following CON or DEP/MS (N=4/group). **a**, RRHO analysis revealed significant concordance between DEP/MS-regulated microglia genes as compared to gene differentials in germ-free adult male mice (Thion et al., 2018) **b**, Significant discordance was observed between DEP/MS-regulated microglia genes as compared to gene differentials in lipopolysaccharide (LPS) treated mice (Hanamsagar et al., 2017). **c**, Significant discordance was observed between DEP/MS-regulated microglia genes as compared to gene differentials across development (P4-P60; Hanamsagar et al., 2017). **d**, Schematic of cross-fostering procedure and subsequent behavioral testing and sample collection. **e**, Neither time spent on the nest, nor licking and grooming (**f**, L+G= licking and grooming) differed between CON and DEP/MS foster dams (N=6/group, un-paired t-tests [CON vs. DEP/MS]: time on nest: p=0.63, L+G: p=0.86). **g**, Pielou’s evenness differed significantly according to condition (N=7–9/group, p=0.01). D → fC males had significantly higher evenness within the cecal microbiome as compared to D → fD males (p=0.009) while evenness trended towards a difference between C → fC and D → fD males (p=0.067) **h**, The composition of the gut microbiome differed significantly according to condition on all four metrics of beta diversity (N=7–9/group, Bray-Curtis: p=0.003, Jaccard Index: p=0.001, unweighted UniFrac: p=0.007, weighted UniFrac: p=0.03, Bray-Curtis shown in PCoA). **I-j**, LEfSe revealed several genera of bacteria that were differentially abundant in DEP/MS-DEP/MS and DEP/MS-CON males including Helicobacter, Parabacteroides, and Bacteroides (N=7–9/group, 1-way ANOVA [treatment], p=0.002, posthoc: D→fD vs D→fC: p=0.007, D→fD vs C→fC: p=0.006, C→fC vs D→fC: p=0.99). **k**, Metabolomic analysis of cecal contents revealed a main effect of treatment such that, relative to control, SCFA concentrations differed significantly in D→fD vs D→fC offspring (N=5/group, p<0.001). In D → fD males, all metabolites differed significantly from C→fC [baseline; single sample t-test] while none of the metabolites did so in D→fC males. **l**, Fostering to a CON dam on the day of birth significantly increased sociability in male DEP/MS offspring (N=7–8/group, unpaired t-test [D → fD vs. D → fC], p=0.005) and restored a preference for the social stimulus (**m**, N=7–8/group, unpaired t-tests [Social vs. Object], D → fD: p=0.70, D → fC p=0.003). Data represent Mean +/− SEM, *p<0.05. CON: vehicle and control, DEP/MS: diesel exhaust particles/maternal stress, p: postnatal, w.: weighted, unw.: unweighted, Rel. Ab.: relative abundance.

The DEP/MS-induced hyper-ramification in microglia that we observe following DEP/MS is like that observed in germ-free mice^25^. This led us to ask whether microglial gene expression also changes in similar ways following DEP/MS and other microbial disruptions. We used strati ed Rank-Rank Hypergeometric Overlap (RRHO) analysis^61^ to compare gene expression changes between two datasets: gene differentials in male microglia following DEP/MS vs. CON (see Fig. 2) to gene differentials in microglia from germ-free vs. conventionally housed male mice in a published dataset^26^. We observed significant concordance between genes that are differentially transcribed following DEP/MS and those that are altered in germ-free microglia (Fig. 5a). We also compared DEP/MS microglial gene differentials to gene differentials following acute immune activation (2h after lipopolysaccharide: LPS at P60^35^, Fig. 5b) and across typical development^35^ (Fig. 5c). Interestingly, we found the opposite pattern (significant discordance) in both comparisons. These findings are in line with the idea that germ-free microglia are immune-incompetent and immature and suggests that a similar phenotype is induced by DEP/MS exposure in male microglia.

### Cross-fostering at birth prevents DEP/MS-induced social deficits in male offspring

Given the established link between gut microbiota and social behavior^62^ we tested whether shifting the gut microbiome towards a CON-typical composition would prevent social deficits in DEP/MS male offspring. Cross-fostering on the day of birth shifts the composition of the offspring gut microbiome towards that of the foster mother^63–65^. DEP/MS exposed pups were fostered to either a different DEP/MS dam on the day of birth (D → fD) or to a CON dam (D→ fC). Similarly, CON exposed pups were fostered to a different CON dam (C → fC) or to a DEP/MS dam (C→ fD). Offspring were then tested on social behavior assays during adolescence prior to sacrifice and sample collection for microbiome and gut analyses (Fig. 5d). We also assessed maternal behavior to rule out that changes we observed were due to differences in maternal care. We found no differences between DEP/MS and CON dams in time spent on the nest nursing (Fig. 5e) or in licking and grooming behaviors (Fig. 5f; Fig. S5).

To verify that cross-fostering of DEP/MS pups to a CON dam shifted the gut microbiome towards a CON-typical phenotype, we used 16S sequencing of cecal contents at P45. Indeed, alpha diversity differed significantly with foster condition (Pielou’s Evenness, Fig. 5g). D→ fC males had significantly higher evenness than D → fD males (Fig. 5g) that did not differ from C→ fC males. Furthermore, PERMANOVA analysis revealed divergent microbial community structure between D → fD, D→ fC, and C→ fC males in all four beta diversity indices (Fig. 5h, Bray-Curtis, Jaccard dissimilarity, unweighted and weighted UniFrac). The microbiome of D → fD males differed significantly from that of C→ fC, and D→ fC males. Linear discrimination analysis effect size (LEfSe) identified several genera of bacteria that differed between D → fD and D→ fC male offspring (Fig. 5i–j). Among these, *Helicobacter*, *Bacteroides* and *Parabacteroides* were more abundant in D → fD male offspring, while *Lachnospiraceae*, and *Oscillospira* were more abundant in D→ fC male offspring. *Helicobacter pylori* (*H. pylori*) has been implicated in gut inflammation and acute gastritis^66,67^. Both *Parabacteroides* and *Bacteroides* are differentially abundant in the gut microbiome of human patients with ASD^68,69^. Neither tight junction protein mRNA nor villi length/mucosal thickness differed between D → fD and D→ fC males in the ileum at P45 (Fig. S6). We also conducted metabolomic analysis of short chain fatty acids (SCFA) to determine whether bacterial metabolites were influenced by our cross-fostering manipulation. We found that 6 SCFAs including acetate and butyrate were increased in D → fD males as compared to C→ fC, and that this increase was abolished in D→ fC males (Fig. 5k).

Newborn offspring acquire microbes from their mother during rearing in the home cage. We hypothesized that DEP/MS-induced changes in the gut microbiome were due to differential transmission of maternal microbes. To our surprise, we found no differences between DEP/MS or CON dams at any timepoint (Fig. S7). Furthermore, there were no differences in the vaginal or milk microbiomes (Fig. S7). These findings point to the intriguing possibility that non-microbial constituents of maternal milk may carry the signal that leads to microbiome restoration in DEP/MS pups fostered to a CON dam, an exciting avenue for future studies.

Next, we tested whether cross-fostering to a CON dam could rescue sociability in DEP/MS-exposed male offspring. As expected, D → fD offspring displayed no preference for a social stimulus (Fig. 5l). However, D→fC males showed significantly higher sociability (Fig. 5m) and spent more time in social investigation as compared to D → fD offspring (Fig. 5l). In the social novelty preference test, D→fC males spent significantly more time in total social investigation as compared to D → fD males, but there was no significant effect on social novelty preference, per se (Fig. S8). This finding may suggest that shifting the gut microbiome increases social motivation across assays, rather than choice of social partner. We also compared the sociability of CON-exposed males fostered to DEP/MS dams (C→ fD) to that of CON-exposed males fostered to CON dams (C→ fC). We found no difference in sociability between these groups, suggesting that cross fostering is insufficient to induce a DEP/MS behavioral phenotype on its own (Fig. S8). In sum, these findings demonstrate that intervening at the level of the microbiome can ameliorate social deficits in male offspring following DEP/MS.

#### Cross-fostering at birth prevents DEP/MS-induced microglial hyper-ramification, but does not affect the dopamine system, in male offspring

**Figure 6:**
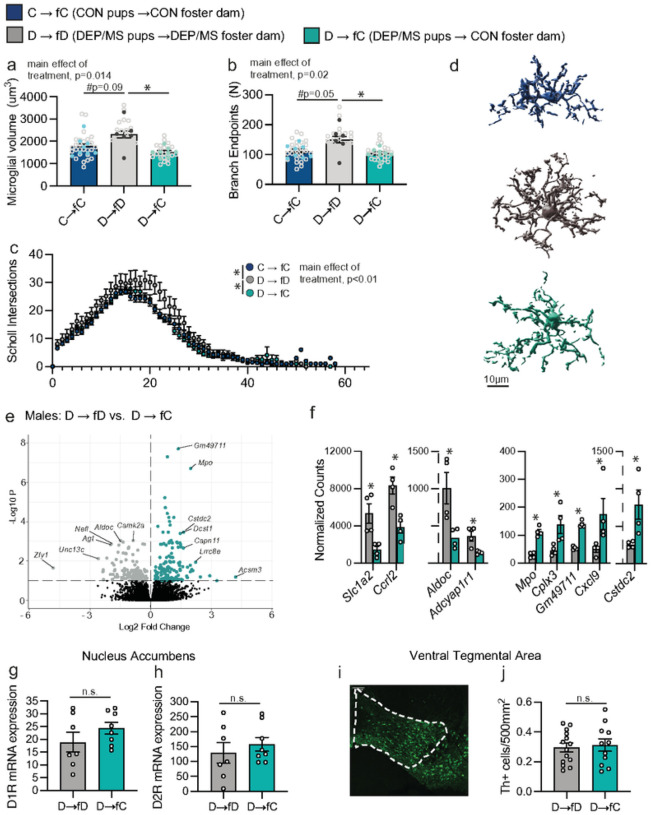
Cross-fostering prevents changes in microglial hyper-ramification but does not alter dopaminergic endpoints. **a**, Imaris 3D reconstruction revealed that cross-fostering to a CON dam at birth reduced microglial volume following DEP/MS (**a**) as well as branchpoints (**b**) and sholl intersections (**c**; N=19–26 microglia from 6–8 animals/group, nested one-way ANOVA [C → fC vs. D → fD vs. D → fC], volume: p=0.014, branch endpoints: p=0.02, sholl intersections: p<0.001). In **a** and **b**, pale grey dots represent individual microglia while darker dots represent animal averages. **d**, Representative reconstructions of CON and DEP/MS microglia, scale=10μm. **e**, Volcano plot of microglia gene expression reveal numerous genes that are differentially expressed in D → fD vs D → fC males at an adjusted p value cutoff of 0.1 (N=4/group, positive Log2 fold change indicates higher expression in D → fC). **f**, Top most significantly altered genes in D → fD vs. D → fC males. **g**, Neither D1 nor D2 (**h**), mRNA expression differed in the nucleus accumbens between D → fD and D → fC males (N=7–8/group, un-paired t-test [D → fD vs. D → fC]; D1R: p=0.23, D2R: p=0.50). **i**, Representative image of ventral tegmental area (VTA) analyzed. **j**, Tyrosine hydroxylase + cell number did not differ in the VTA between D → fD and D → fC males (N=7/8/group, un-paired t-test [D → fD vs. D → fC], p=0.73). Data represent mean +/− SEM *p<0.05.

Finally, we asked whether cross-fostering to a CON dam prevented the microglial and dopaminergic phenotypes we observed in DEP/MS-exposed male offspring. Using Imaris 3D image reconstruction, we observed significant main effects of treatment on microglial volume (Fig. 6a), branch endpoints (Fig. 6b) and Sholl intersections (Fig. 6c). D→ fC males had significantly smaller and less ramified microglia as compared to D → fD males (Fig. 6d), indicating that microbial intervention at birth prevents microglial, as well as social, alterations. We also conducted RNA sequencing of NAc-microglia isolated from D → fD and D→ fC males. Gene expression differed dramatically between these two conditions, with many microglial genes both up- and down-regulated following cross-fostering to a CON dam at birth (Fig. 6e). Interestingly, these genes were not the same gene sets that differed between CON and DEP/MS males in our previous assessment. Rather, genes such as *Ccrl2*, *Cxcl9*, *Mpo*, and *Cstdc2* were the most up or down regulated (Fig. 6f). These changes suggest, not surprisingly, that a distinct transcriptional profile is associated with returning microglia to a CON morphological phenotype following DEP/MS (D→ fC) compared to those that differed between CON and DEP/MS independent of cross-fostering. Thus, cross-fostering, on its own, likely shifted the microglial transcriptome. We also assessed D1 and D2 receptor mRNA within the NAc, as well as Th cell number within the VTA, to determine whether cross-fostering to a CON dam prevented the changes we observed in the dopamine system. Interestingly, we found no difference between D → fD and D→ fC males in D1R mRNA (Fig. 6g), D2R mRNA (Fig. 6h), or Th cell number (Fig. 6i&j).

## Discussion

While epidemiological work has incontestably shown that toxicants and psychosocial stressors converge on vulnerable populations to increase disease burden, little mechanistic work has been done to understand the underlying biological substrates of these effects. Here, we show that DEP/MS alters the composition of the gut microbiome, induces microglial hyper-ramification, and decreases dopaminergic tone within reward circuits in offspring, all in a male-biased manner. Furthermore, we find novel evidence that social behavior deficits following DEP/MS can be rescued or prevented by activating the dopamine system or by shifting the composition of the gut microbiome. Moreover, modulation of the gut microbiome at birth prevents changes in microglial morphology, but not changes in the dopamine system.

Changes in social behavior, microglia, and the dopamine system have been reported in models of maternal immune activation (MIA) that use bacterial and viral mimetics (i.e. lipopolysaccharide [LPS] and Poly I:C, respectively) to directly activate the maternal immune system^71, 72^. Our findings suggest that gestational exposure to air pollution and maternal resource deprivation can elicit similar phenotypes in offspring – highlighting the harmfulness of these pervasive environmental exposures. This is especially interesting given that the diesel exhaust particles are given to the mother, and yet they have a potent, enduring impact on offspring. Our previous work showed that DEP/MS exposure leads to elevated maternal blood serum concentrations of cytokines such as IL-6, IL-17a, and TNFα, similar to what is observed in MIA^32^. This may suggest that DEP/MS alters the postnatal microbiome offspring as a secondary consequence of fetal reprogramming of the gut and brain by maternal immune factors.

Interestingly, while previous work in several ASD models has shown that modulation of the gut microbiome acts via the dopamine system to Influence behavior^27,28^, we find that restoration of the gut microbiome does not alter the dopamine system. Rather, we find that microglial morphology is robustly impacted by microbiome modification. This is in line with previous work showing that the microbiome is a potent organizer of microglial development, particularly during the perinatal period. For example, ceasarean-section delivery – which prevents offspring from receiving microbes typically acquired during passage through the vaginal canal – and represents a highly stressful birth event – leads to altered microglial colonization in the hippocampus and paraventricular nucleus of the hypothalamus^73^.

Importantly, brain region-specific modification of dopaminergic function remains difficult in human patients. In contrast, the gut microbiome represents a more tractable therapeutic target. One strength of our work is that our cross-fostering procedure demonstrates that shifting the overall composition of the gut microbiome is effective at restoring sociability. There are several possible mechanisms by which this might be mediated, including changes in microbial metabolites such as short chain fatty acids (SCFAs) and direct vagal nerve activation^62^. Indeed, our findings show that several SCFAs such as acetate and butyrate may be important mediators. Future studies are needed to determine the precise route by which changes in the gut microbiome following DEP/MS exposure leads to changes in the brain and, therefore, social behavior.

One important point is that while modification of the gut microbiome is sufficient to prevent social behavior impairments in male offspring following DEP/MS, cross-fostering to a DEP/MS dam was insufficient to induce social behavior deficits in CON males. Excitingly, this suggests that the gut microbiome may be an important target for intervention, even if not responsible for the entire disease phenotype. Similarly, Sgritta et al., (2019) found that supplementation with *L. reuteri* rescued social behavior deficits in valproic acid-treated mice, despite *L. reuteri* abundance not being reduced by this exposure in the first place. It also suggests that changes to the immune system and/or intestinal epithelium in utero may precede, and to some degree dictate, which bacterial taxa gain a foothold. This is especially likely given that our effects are observed in male but not female offspring, despite the same microbiome exposure. Indeed, numerous studies have reported sex differences in the gut microbiome and microglia, as well as their relationship to each other^26,74^. For example, Thion et al. characterized microglial brain colonization and gene expression in male and female germ-free vs. conventionally housed mice. They found that germ-free male mice had significantly altered microglial colonization and gene expression during embryonic development, while female germ-free mice had more microglial transcriptional changes in adulthood^26^. Elucidating the mechanisms underlying such sex differences is an important area for future investigation.

In closing, our results characterize male-specific behavioral, microglial, and neural effects of synergistic exposure to both air pollution and maternal stress during pregnancy. Furthermore, we identify two potential sites for therapeutic intervention in the treatment of social behavior impairments in ASD.
